# Soft resonator of omnidirectional resonance for acoustic metamaterials with a negative bulk modulus

**DOI:** 10.1038/srep16110

**Published:** 2015-11-05

**Authors:** Xiaodong Jing, Yang Meng, Xiaofeng Sun

**Affiliations:** 1 Fluid and Acoustic Engineering Laboratory, School of Energy and Power Engineering, Beihang University, Beijing 100191, China

## Abstract

Monopolar resonance is of fundamental importance in the acoustic field. Here, we present the realization of a monopolar resonance that goes beyond the concept of Helmholtz resonators. The balloon-like soft resonator (SR) oscillates omnidirectionally and radiates from all parts of its spherical surface, eliminating the need for a hard wall for the cavity and baffle effects. For airborne sound, such a low-modulus resonator can be made extremely lightweight. Deep subwavelength resonance is achieved when the SR is tuned by adjusting the shell thickness, benefiting from the large density contrast between the shell material and the encapsulated gas. The SR resonates with near-perfect monopole symmetry, as demonstrated by the theoretical and experimental results, which are in excellent agreement. For a lattice of SRs, a band gap occurs and blocks near-total transmission, and the effective bulk modulus exhibits a prominent negative band, while the effective mass density remains unchanged. Our study shows that the SR is suitable for building 3D acoustic metamaterials and provides a basis for constructing left-handed materials as a new means of creating a negative bulk modulus.

Resonance is of fundamental importance in the acoustic field, particularly to the manipulation of sound waves within a distance far smaller than wavelength. Built-in local resonance can endow composites that are made of subwavelength resonators with unusual dispersions, ranging from exponential decaying to negative refraction. Such artificially designed composites are known as acoustic metamaterials because they can break the conventional rules of sound wave propagation in ordinary materials^1^,^2^,^3^,^4^,^5^,^6^,^7^,^8^,^9^. When the composites are treated as homogenous media, their properties correspond to a medium with negative effective constituent parameters. Based on the concept and methodology of negative parameters, the research of acoustic metamaterials has been continuously progressing towards extraordinary applications, such as super acoustic damping or insulation, acoustic hyperlens, acoustically invisible cloaks and so on^10^.

Resonance can be characterized by its spatial symmetry of oscillation. Monopolar and dipolar resonances are the two basic forms of resonance, with rotational and polarizing symmetry, respectively. Moreover, in the context of acoustic metamaterials, the spatial symmetry of resonance has special implication in creating negative effective constituent parameters. A negative mass density or bulk modulus, albeit impossible for static situation, can occur as a result of out-of-phase oscillation in a frequency band above the resonance. In the out-of-phase regime, resonance with rotational symmetry causes dynamic volume change at the same sign as the pressure change, whereas normally, the two are of opposite signs, leading to a negative bulk modulus. When the symmetry of resonance is polarizing, however, a negative mass density occurs in the sense that the mass center accelerates in the opposite direction of the driving force. As a result, it is the symmetry that determines which of the two basic constitute parameters becomes negative on the macroscopic scale.

In recent years, the study of acoustic metamaterials has been strongly stimulated by the advent of new types of resonators, such as the rubber coated sphere^1^ and the mass-decorated membrane^2^. These resonators are dipolar resonators and serve to realize negative mass density. On the other hand, a negative bulk modulus results from the mechanism of monopolar resonance with rotational symmetry. Natural or man-made bubbles can resonate with monopole symmetry at the best approximation^11^, but the bubbles are restricted to an underwater environment. So far, in terms of the realization of the negative bulk modulus, researchers have mainly resorted to the use of Helmholtz resonators (HRs)^3^,^4^,^5^,^6^,^7^,^8^. Strictly speaking, monopole symmetry can only be satisfied in the sense of spatial average over an HR. The resonant structure is usually described as bottle-like, with the fluid slug in the neck(s) acting as a mass and the volume of the cavity functioning as a spring. The hard wall of the cavity is essential to provide a baffle effect. Without the baffle, the oscillating fluid slug, just like a vibrating suspended disk, radiates in a dipolar pattern^12^. As both its inductance and capacitance are derived from a single fluid, an HR can solely be tuned by adjusting its geometry. Thus, the resonator may become impractically large when dealing with low-frequency sound. Currently, despite the numerous modifications to HRs, there has been limited success to experimentally achieve a negative bulk modulus. More attention has focused on the anomalous propagation in one-dimensional (1D) tunnels^3^,^6^,^7^ or planar (2D) waveguides^8^ with shunted HRs. In short, Helmholtz resonators represent a category of volume-source-like acoustic resonators characterized by partial openings in a hard-wall cavity or a cluster of cavities. Evidently, we need to go beyond this conventional concept and achieve subwavelength monopolar resonance using a new approach, which is particularly important to the development of acoustic metamaterials and is also of general interest to the acoustic field. More recently, efforts have been pursued to realize monopolar resonance in a different manner from the classical HRs, in particular, the resonating unit formed by a couple of mass-decorated planar membranes and a solid ring^13^.

The primary purpose of this paper is to design and realize a type of soft resonator (SR) with spherical oscillating symmetry. In our work, the basic ideas of the SR are exemplified with two types of popular toys. Using experimental results and theoretical analyses, we demonstrate that the SR is capable of producing near-perfect monopolar resonance and that it has some unique features.

## Results

### Concept and theory

**Figure 1:**
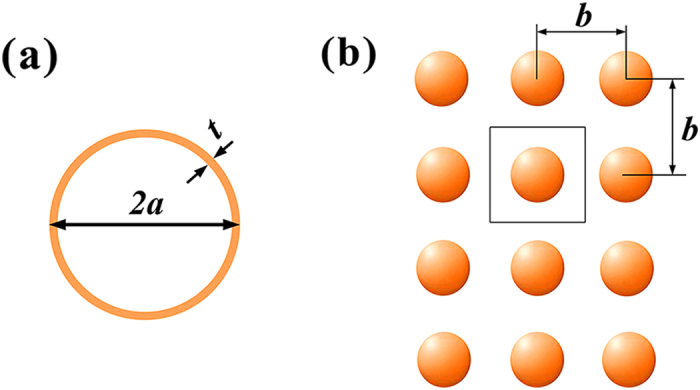
(**a**) Basic structure of a soft resonator. (**b**) A crystal-like lattice of SRs with a lattice constant of *b*, where the line square indicates the unit cell defined by the first Brillouin zone for the field averaging analysis.

As illustrated in Fig. 1(a), the SR we propose is as simple as a spherical shell with encapsulated gas. The outer shell is highly soft, which can be made of flexible materials, such as latex. Similar to the surface tension of a water drop, there is a slight elastic tension in the shell that keeps the shape of the SR spherical. In analogy to an LC electrical circuit, the soft shell is nearly a pure inductor at low tension, while the encapsulated air acts as the capacitor. Gas with a smaller specific heat ratio, such as helium, is an alternative to air due to the benefit of increasing the capacitance without increasing the volume^14^. Figure 1(b) shows a slab of acoustic metamaterial made of an SR lattice in air.

Using the electrical-acoustic analogy, we formulated the acoustic impedance of a unit SR with lumped element modeling, as described below:


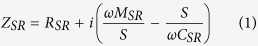


where 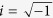, *ω* is the angular frequency, and *S* is the surface area of the spherical shell; the acoustic resistance, 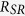, is the sum of the viscous resistance, 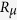, and the radiation resistance, 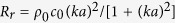
11, where *k* is the wavenumber; the acoustic inductance, 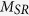, includes the shell mass, *M*, and the radiation mass, 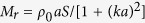
11; the acoustic capacitance is 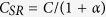, where 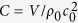 (*V* is the gas volume) and 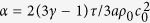 accounts for the effect of shell tension *τ* 11; and 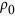, 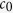 and *γ* are the density, sound speed and specific heat ratio of air, respectively. The resonance occurs when the linear frequency part balances with the inverse frequency part in Eq. (1); thus, the angular resonant frequency is written as


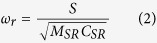


**Figure 2:**
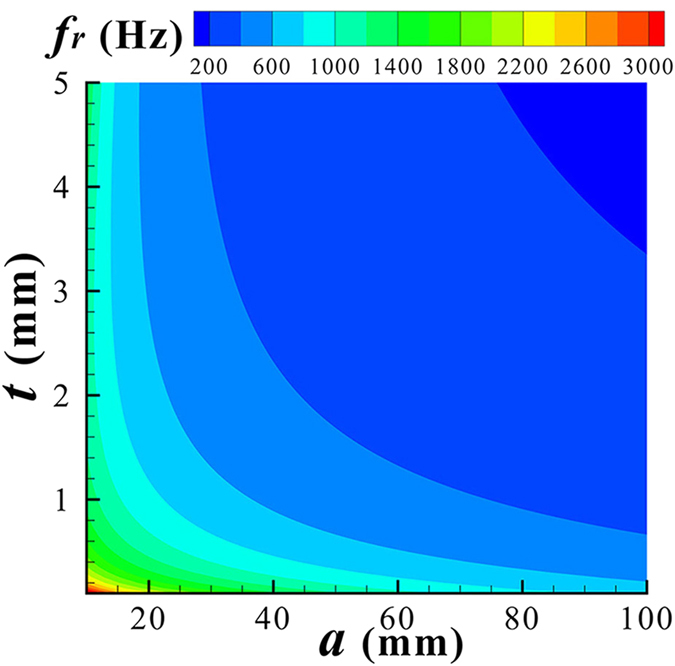
Contour plot for the resonant frequency of a unit SR as a function of outer radius (*a*) and shell thickness (*t*) calculated from Eq. (2).

The above formula indicates that the resonant frequency of an SR depends on the outer radius, the shell thickness and the membrane tension. Generally, increasing the shell tension leads to the increase of the resonant frequency because the tightening effect is equivalent to reducing the gas volume by a ratio of 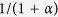. However, this effect is weaker compared with those of the gas volume and shell mass because, generally, a small membrane tension is sufficient to retain the spherical shape of the SR. Neglecting the effect of *τ*, it can be deduced from Eq. (2) that the resonant frequency is proportional to 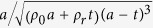, where 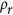 is the density of shell material. The contour plot in Fig. 2 shows the resonance frequency as a two-dimensional function of SR radius, *a*, and shell thickness, *t*, assuming the shell material is latex. As shown in Fig. 2, an SR as small as 1–2 centimeters in radius with a thin shell less than 1 mm can work in the frequency range of 1.0–3.0 kHz. For a resonance below 500 Hz, an SR necessitates a relatively larger radius amounting to several centimeters and a thicker shell of a few millimeters.

For an acoustic metamaterial made of subwavelength units, homogenized medium theory is appropriate for analyzing its response to an incident sound wave. The electrical-acoustic analogy, which is usually applied to lumped element modeling, can also be extended to the field averaging analysis. Following the field averaging method for obtaining effective parameters of the electromagnetic composites in^15^, our analysis considers a unit cell defined by the first Brillouin zone, as shown in Fig. 1(b). The homogenization implies that the material response is equivalent to that of a fluid-like medium. For such a medium in the unit cell, the integral form of the time-harmonic linearized continuity equation is written as


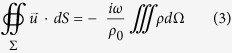


where ∑ and Ω denote the surface area and the volume of the unit cell, respectively, and 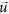 is the fluctuating velocity over ∑. The above equation relates the acoustic density to the fluctuating volume flux stemming from two sources: the pulsation of the SR and the compressibility of the fluid outside the SR. The normal pulsating velocity on the shell surface can be calculated from the definition of the specific acoustic impedance, 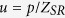, where 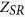 is given in Eq. (1). The pressure rise is uniform over the length scale of the unit cell. Thus, we can obtain the average acoustic density:


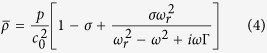


Next, substituting Eq. (4) into the isotropic relation 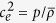, we arrive at the equation of the effective sound speed:


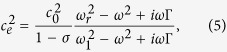


where 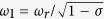; *σ* is the filling ratio of the SRs in the lattice; and 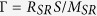 is a loss-related parameter. Due to the omnidirectional oscillation of the SR, the mass acceleration of the shell contributes little to the effective density. Thus, the effective bulk modulus is written as follows:


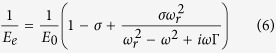


where 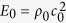 is the bulk modulus of the ambient air.

For a plane wave normally incident on a slab of homogenized metamaterial, the amplitude transmission coefficient can be calculated with the effective parameters^5^, as shown below:


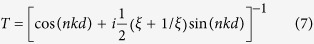


where *d* is the slab thickness, 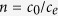 is the relative refractive index, and 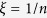 is the relative acoustic impedance. In deriving the above equations, the time dependence of 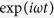 is assumed.

### Experiment

**Figure 3:**
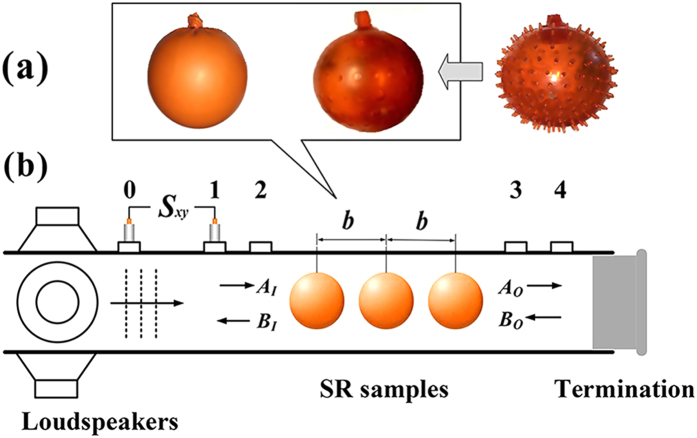
(**a**) Images of the two types of SR samples: small balloons (*t* = 0.21 mm, 2*a* = 42 mm) and rubber balls with thorns removed (*t* = 1.0 mm, 2*a* = 45 mm) together with the original one with thorns, where the shell thickness and the diameters are average values. (**b**) Schematic of the square waveguide setup for measuring the scattering matrix of the SR samples using the two-load method, where a single movable microphone at port 1, 2, 3 or 4 and a fixed reference microphone at port 0 are used to obtain the complex acoustic pressures from the cross-spectrum (*S*_*xy*_) between their output signals.

In the experiment, the proof-of-concept SRs are conveniently obtained from two types of popular toys, i.e., small balloons and rubber balls with thorns, as shown in the images in Fig. 3(a). The small balloons are denoted as SRs-A. To produce a second sample, SRs-B, the thorns were removed from the toy balls. Therefore, the thorns play no role in the experiment. Both samples are inflated with air. The geometrical parameters of the two samples are as given in the legend of Fig. 3. The shell tension is determined from the Young-Laplace law, 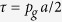, where the inflation gauge pressure, 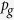, is measured by inserting the probe of a small pressure meter into the samples.

The acoustic measurement is performed in a square waveguide, where the samples are arranged in one row along the axial direction. For the situation of a plane wave normally incident on the cubic lattice in Fig. 1(b), we can consider a single row of the SRs due to the symmetry of the SR and the periodicity condition between adjacent rows. As shown in Fig. 3(b), the waveguide is a 1.5-m-long tube with a square cross section of 50 × 50 mm^2^ and is made of acrylic material. The test section is in the axial middle of the waveguide, where the portion of upper wall can be removed for installing the SR samples into the waveguide. The samples are located in the test section by simply hanging from the upper tube wall. The spherical outer surfaces of the samples are separated from the inner walls of the waveguide by a gap of approximately 2.5–4.0 mm (at the closest point), such that the elastic shells are free of oscillation. In principle, a matrix material is dispensable for an SR to produce monopolar response; therefore, we did not introduce a matrix material for holding the samples together.

The sound source is comprised of two pairs of face-to-face loudspeakers on one end of the waveguide. A digital control method is used for the loudspeakers to generate multi tones with nearly constant amplitude^16^. To ensure only plane waves exist in the waveguide, the upper tonal frequency is kept below 3.0 kHz, which is slightly lower than the cutoff frequency of the first nonplanar mode. There is a pair of microphone ports in front of and behind the test section. The complex sound pressures are measured by a single movable microphone installed at each of the four ports and a reference microphone fixed near to the sound source. A cross-spectrum analysis is performed between the signals of the movable microphone and the reference microphone to determine the phase of the sound pressures. The single-microphone technique can avoid measurement errors that are associated with microphone phase mismatch in the harmonically static situation^17^. The use of multi-tone source signal enables the acquired sound pressure spectrum to have a fine resolution of frequency (10 Hz).

The two-load method^18^ is used to obtain the scattering matrix of the slab composed of a single row of the samples in the test section. Two loads imply that there are two different termination conditions on the end of the waveguide opposite to the sound source. The sound pressure measurement is taken twice: once for the open exit and once for the highly reflective condition imposed by a small absorptive plug. For each load or termination condition, the sound pressure data acquired at the front pair of the ports (1 and 2 in Fig. 3(b)) is used to decompose the sound field in front of the test section into the forward and backward modes by means of the standard two-microphone transfer function method^16^. The wave decomposition is also performed for the sound field behind the test section. Assuming that the sound transmission through the samples is reciprocal, there is a general relation for the acoustic modes in the waveguide: 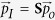 with 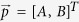, where **S** is the 2 by 2 scattering matrix; *A* and *B* are the complex amplitudes of the forward and backward modes, respectively; and the subscripts *I* and *O* denote the modes in front of and behind the test section, respectively, as shown in Fig. 3(b). This relation can be written for each of the two loads; thus, four linear equations are formed to solve the unknown elements of **S**. Then, both the transmission and reflection coefficients can be calculated from the scattering matrix^18^.

### Comparison of theoretical results with experimental data

**Figure 4:**
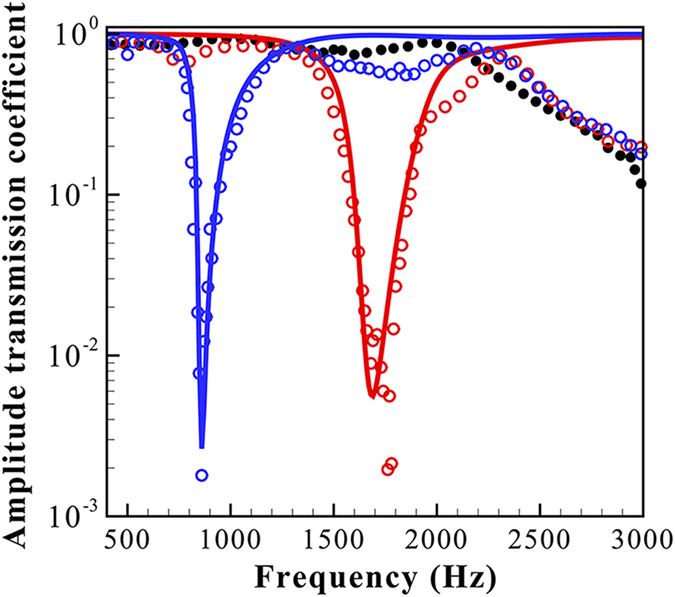
The experimental transmission coefficients for the SRs-A (red circles) and -B (blue circles) are presented as a function of frequency, showing excellent agreement with the predictions (solid line) of Eq. (7). The band gaps for the two samples occur at 1700 Hz and 850 Hz, respectively. For the results of the water-filled balloons (solid circle), the transmission decrease is due to the Bragg scattering effect as frequency approaches 3.0 kHz.

As shown in Fig. 4, for a 150-mm-thick slab composed of 3 SRs-A or -B in one row (*b* = 50 mm), the transmission coefficients reach lower than 0.5% at the dips of certain narrow bands. Thus, the incident acoustic energy is almost entirely (>99.999%) blocked from transmitting at the band gaps. This result is very significant, considering that the SRs are extremely lightweight and sparsely distributed (corresponding to a 30% filling ratio in the lattice). Moreover, the band gaps occur at subwavelength scales, with 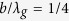 and 1/8 for the SRs-A and -B, respectively, where 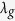 is the wavelength at the band gap frequency. As a comparison, we also measured the small balloons of 42 mm in average diameter filled with incompressible water and did not observe similar transmission dips. Although the Bragg scattering is shown to cause the decrease in sound transmission, this decrease occurs near 3.0 kHz, where the wavelength matches the lattice constant. Evidently, the subwavelength band gaps result from the local resonances of the SRs. The predicted resonant frequencies using Eq. (2) are 1680 Hz and 850 Hz for the SRs-A and -B, respectively, which agree with the experimental frequencies of the band gaps. Thus, by increasing the shell thickness or equivalently the shell mass (the material of both toys is latex), the resonant frequency of an SR can be considerably reduced.

Figure 4 shows that the amplitude transmission coefficients predicted by Eq. (7) are in excellent agreement with the experimental results, in terms of both the shapes and magnitudes of the transmission curves. When the frequency approaches 3.0 kHz, the deviation occurring between the predictions and the experimental results is understandable, given that the Bragg scattering effect is not taken into account by the homogenized medium theory.

**Figure 5:**
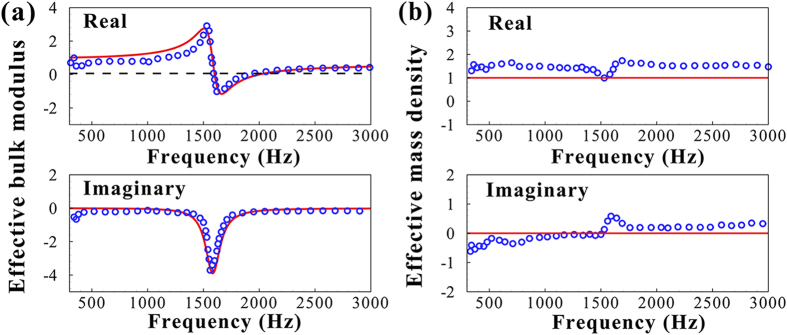
The experimental (blue circle) effective parameters are plotted as a function of frequency for a 50-mm-thick slab composed of one SR-A, showing excellent agreement with the predictions (solid line) of Eq. (6). (**a**) The effective bulk modulus (normalized by 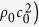 is frequency-dependent, with a narrow band of negative real part above resonance. (**b**) The effective mass density (normalized by 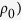 exhibits a nearly constant real part approximately equal to 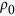 and a near-zero imaginary part.

The effective bulk modulus, *E*_*e*_, is retrieved from the measured transmission and reflection coefficients with the standard retrieval method^19^. Figure 5(a) shows the results for a 50-mm-thick slab composed of only one SR-A in the waveguide. Excellent agreement is achieved between the predictions and the experimental results for both the real and imaginary parts of *E*_*e*_. There is a prominent region of negative *E*_*e*_ (real part) on the higher frequency side of the resonance, showing the merit of the SR as a new means to produce the negative effective modulus. As further evidence of the monopolar symmetry of the SR’s oscillation, it is demonstrated that the measured effective mass density of the slab is almost equal to the ambient air density over the entire frequency range in Fig. 5(b), where the small jumps in the results near the resonance are probably due to the minor asymmetries in the shape or mass distribution of the small balloon. To our knowledge, there is still a lack of such experimental results on the effective mass density for proving the symmetry of the resonance in the previous studies for the HRs. Within a narrow band above resonance, the shell motion becomes out of phase with respect to the applied sound pressure, i.e., expanding under positive pressure and compressing under negative pressure. When the SRs form a crystal-like lattice, from a microscopic point of view and in analogy to Fano-type interferences^20^, their out-of-phase energy-releasing oscillations give rise to collective radiation, leading to either cancellation of the incident wave (exponentially damping) or steering of the acoustic energy flow (wave bending).

## Discussion

The concept of SR is essentially different from the conventional designs of HR and its variants. When an SR is submitted to an isotropic acoustic pressure, all parts of its spherical shell oscillate uniformly in all directions, thus eliminating the need of a hard wall for the cavity and baffle effects. Moreover, the SR can be efficiently tuned by adjusting either the thickness or the material density of the shell. This advantage is also distinct from the natural bubbles. Deep subwavelength resonance is achieved, benefiting from the large density contrast between the solid shell material and air. The sample with a 1-mm-thick shell resonates at 850 Hz, corresponding to a wavelength 9 times longer than its diameter. Certainly, the wavelength to diameter ratio can be further increased by increasing the shell mass or using helium as the encapsulated air. Another feature of the SR is that there is little viscosity-related loss, making it more apt for low-loss applications. We can also observe the difference between the designs of the rubber coated sphere^1^ and the present SR, except that the former is for dipolar resonance. The outer soft shell of the SR acts as the mass instead of the inner hard sphere, and the encapsulated gas serves as the spring, which is much softer than the available rubbers, provided that the gas volume is not too small. As a result, by eliminating hard massive inclusions and using low-modulus encapsulated air as the spring, the SR can be made extremely lightweight. For a lattice of SRs, a band gap blocks nearly all the incident acoustic energy (>99.999%) within a narrow frequency range. The effective bulk modulus is found to be frequency dependent and has a prominent negative band, while the effective mass density remains approximately the same as that of the ambient air. The experiment and the homogenized medium theory are in excellent agreement, well demonstrating the monopolar characteristics of the SRs. Note that this work only exemplifies the essential features of the SR by conveniently using everyday life toys; therefore, we anticipate that variants can evolve from these basic ideas in future studies. Although the proof-of-concept experiment was performed in the 1D waveguide setup, it is evident that the SRs can act as the building blocks of 3D acoustic metamaterials. Moreover, because the hybridization between monopolar and dipolar resonances is the key to achieving double-negative parameters^21^,^22^, the SRs also provide a basis for designing novel left-handed materials for a wide range of acoustic applications. Surely, the SRs can also be used for conventional purposes as acoustic resonators, especially when omnidirectional response is preferred. As an interesting possibility, the decoration balloons may be used as an environmental noise shield as well. However, for noise abatement applications, the SR has the limitation of narrow-band effectiveness, similar to most of the local resonant units. One method to achieve broad-band effectiveness is by stacking differently tuned SR units into a multilayer panel, as previously demonstrated for the membrane-type metamaterials^23^. This method, however, must be realized at the cost of increasing panel thickness. The latest design of membrane-covered honeycomb metamaterial shows a new possibility to achieve sound transmission loss in the low frequency range^24^. But it is still challenging to develop thin-layer broadband acoustic metamaterials based on the concept of negative material parameters.

## Methods

### Experimental devices

In the experiment, the microphones used are1/4 in. pressure field B&K 4938-L-002 microphones. The outputs of the microphone conditioner are sampled using the National Instruments NI USB-6259 DAQ data acquisition board.

### Physical parameters

In addition to the geometrical parameters of the SR samples, the other measured inputs for the computation include room temperature of 22 °C, air pressure of 101154 Pa, latex density of 860 kg/m^3^, and shell tension of 40 N/m and 26 N/m for SRs-A and -B, respectively. For the viscous resistance, 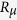, a small value of 0.15–0.3 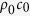 is empirically adopted.

## Additional Information

**How to cite this article**: Jing, X. *et al.* Soft resonator of omnidirectional resonance for acoustic metamaterials with a negative bulk modulus. *Sci. Rep.* **5**, 16110; doi: 10.1038/srep16110 (2015).

## Supplementary Material

Supplementary Information
