# Supplementary material for: Soft resonator of omnidirectional resonance for acoustic metamaterials with a negative bulk modulus
Source: Sci Rep. 2015 Nov 5;5:16110. doi: 10.1038/srep16110 (PMC4633608; doi:10.1038/srep16110)
Supplement: Supplementary Information [file srep16110-s1.doc]

**Supplemental materials for**

**Soft resonator of omnidirectional resonance for acoustic metamaterials with a negative bulk modulus**

Xiaodong Jing, Yang Meng and Xiaofeng Sun

Fluid and Acoustic Engineering Laboratory, School of Energy and Power Engineering, Beihang University, Beijing 100191, CHINA

1. **The prepared SR samples**


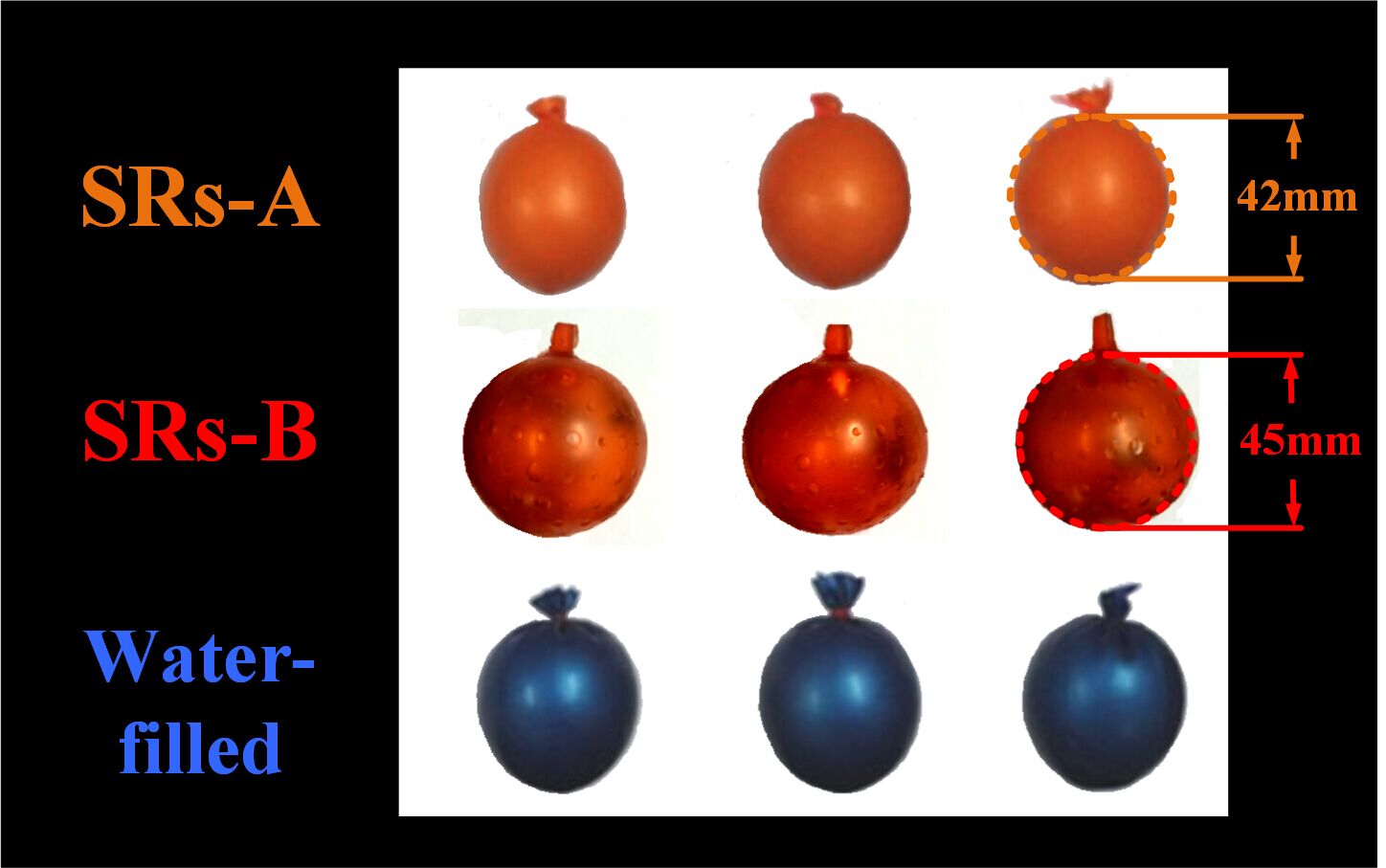


1. **Measurement of the inflation pressure of an SR using a small pressure meter**


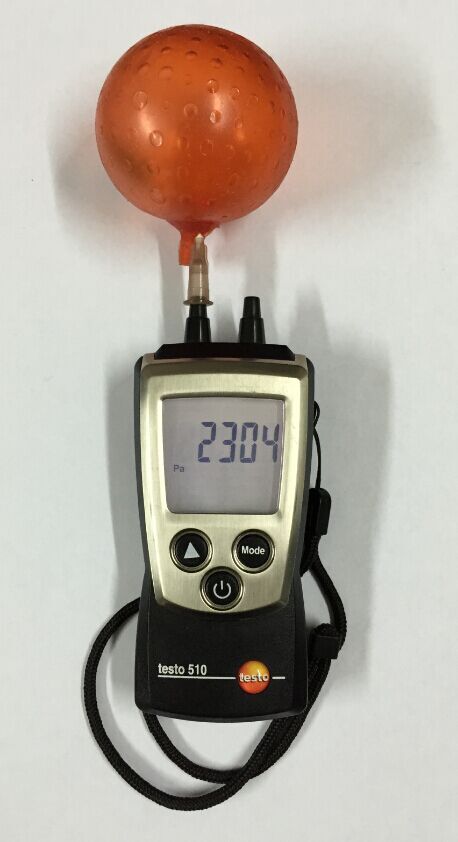


1. **Image of the experimental setup**

**
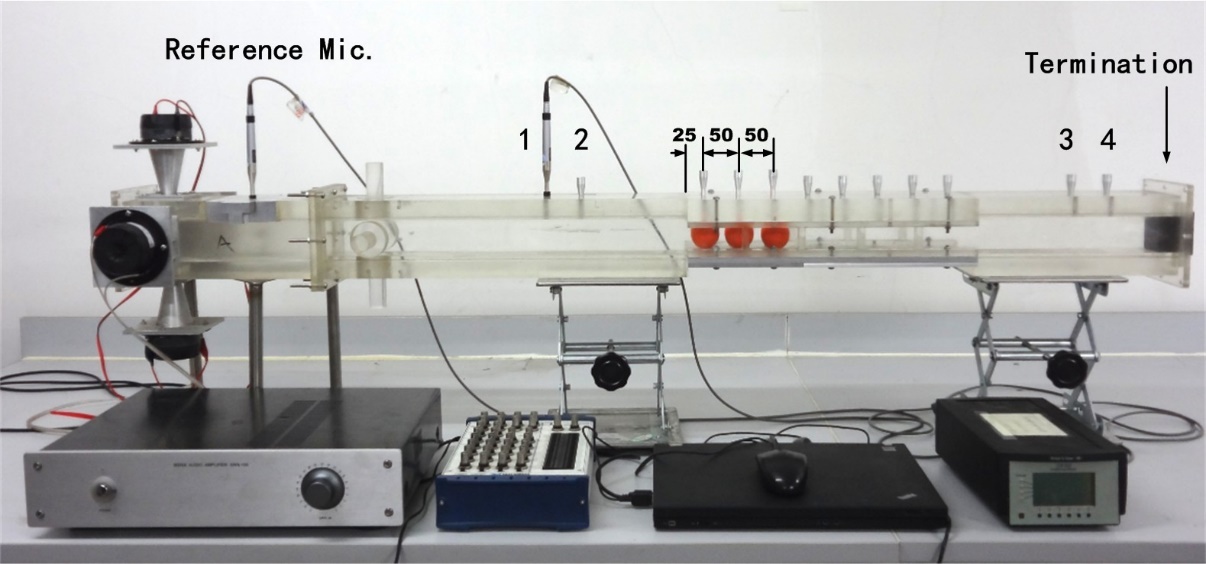
**
